# Copernicus Data Space Ecosystem establishes public cloud processing for earth observation data

**DOI:** 10.1038/s41597-026-06765-8

**Published:** 2026-02-26

**Authors:** Dávid D.Kovács, Jan Musial, Jędrzej Bojanowski, Dennis Clarijs, Jurry de la Mar, András Zlinszky

**Affiliations:** 1 Department of Geodesy and Geoinformation, TU Wien, Wiedner Hauptstrasse 8, Vienna, 1040 Austria; 2 CloudFerro S. A. Fabryczna 5, Warszawa, 00-446 Poland; 3 VITO Remote Sensing, Boeretang 200, Mol, 2400 Belgium; 4 T-Systems International GmbH, Deutsche-Telekom-Allee 5, Darmstadt, 64295 Germany; 5 Sinergise Solutions d.o.o., Cvetkova Ulica 29, Ljubljana, 1000 Slovenia

**Keywords:** Environmental impact, Scientific data, Software

## Abstract

The Copernicus Data Space Ecosystem is the official data platform for the Copernicus Programme’s satellites. CDSE combines instant access to satellite imagery with Application Programming Interfaces and virtual machine processing. Instead of downloading satellite imagery for local computation, CDSE utilizes cloud-optimized files to provide data according to the filtering and processing request of the user, facilitating large-scale scientific analysis. Cloud computing on CDSE eliminates the need for users to rely on their own data infrastructure. The incorporated standards support both Open Science and commercialization of scientific tools and algorithms. CDSE serves all users from beginners to professionals, from the interactive visualization of imagery to custom ML algorithms. Acquiring the skills required to process Earth Observation data is facilitated by the open-source codebase and tutorials. Access to public cloud processing is expected to foster the uptake of Earth Observation across new domains. CDSE now provides the critical mass to serve as a tool for knowledge exchange and to influence commercial and public providers alike to support cloud processing.

## Introduction

In the era of rapid environmental changes and increasing demand for sustainable solutions, access to reliable, long-term and global climate information has never been more critical^1^. Addressing these issues, the European Union’s (EU) Copernicus Programme monitors the Earth’s environment with information services based on satellite imagery, *in situ*, and modelled data^2^. The programme focuses on six thematic areas: Atmosphere, Marine, Land, Climate Change, Emergency Management, and Security^3^. The Copernicus Services provide inputs for the EU’s initiative on Evidence Based Policy^4^ through reliable, quality-assured data in near real-time. In recent years, IT technologies from other fields have been adopted in remote sensing, leading to notable progress in both research and operational applications. The surge in data volumes generated by Earth Observation (EO) satellites has driven the growth of cloud computing architectures, and modern web technology now enables easier access to remote sensing data^5^. Until early 2023, Copernicus data was available to the public through the Copernicus Open Science Hub. This portal had a data viewer with search functionality and supported APIs that allowed users to query the image database and download data for a study area as specific granules. When a specific area was searched, the system returned all complete granules that intersected the area of interest, rather than only the user defined subset. After downloading to local machines, the user created the spatial, temporal or spectral subset of the data, which was memory and labour-intensive job. In fact, the difficulty and resource-intensiveness of data access created a bottleneck, leaving many vulnerable parties unable to make the most of the data offered by the Copernicus Programme^1^.

Cloud processing capacity was previously offered by a network of Data and Information Access Services (DIAS-es): e.g.: CREODIAS, Mundi, ONDA, Sobloo, and WEkEO^6^. These were web-enabled cloud data storage and processing hubs that could serve the imagery directly to the users or to their own virtual machines, where users could perform data analytics. DIAS-es were typically designed to operate in a public-private partnership, established mainly from public funding but with the intention to operate their services on the market. Some of these aimed to be specific to a certain domain or application, others were limited to a certain region or country. Nevertheless, many companies, institutions and national agencies decided to build and operate their own EO data storage and processing platforms, tailored to their requirements^7^. These include the platforms such as the French Theia^8^, CEDA from the UK^9^, and the German CODE-DE^10^. The reasons to do this include compatibility with existing pipelines, application of selected pre-processing tools or additional datasets or very high-resolution imagery, or close integration of data storage and security^11^. With these solutions, stakeholders could operate their bespoke computing solutions. However, this introduced significant redundancy, e.g. for the case of Hungary, a national DIAS has been established, an international and regional data cube platform is operational (Danube Data Cube), but nevertheless, several institutions and companies are operating their own data hubs covering the whole country. This highlights the need for a centralised, generic yet flexible solution for EO data distribution^12^.

As the Copernicus Programme continuously evolves, the volume of data and the necessary computational resources are increasing exponentially^13^. Processing of EO data at the extent of dozens of square kilometres may still be carried out on a local workstation, but operational data analysis at regional-to-continental scale needs scalable cloud-based processing. ML and AI algorithms increase this demand even further^14^. Processing data stored remotely on virtual machines in the cloud - accessed through online interfaces - is rapidly becoming the standard approach for most big data tasks^15^. The evolution of cloud storage technology has also changed the paradigm of data access: instead of downloading in pre-defined units, datasets are now streamed according to the demands of the processing algorithm. Unpacked, cloud-optimized file formats are a major step forward, since they support rapid subsetting of the data using byte-range HTTP requests without reading the whole file^16^. This is making data mirrors redundant: accessing data from a cloud storage located several hundreds of kilometres away is not significantly slower than accessing a network drive that is physically located next door^17^.

In addition to advances in data storage technology, the evolution of widely recognized EO standards - such as the SpatioTemporal Asset Catalog (STAC)^18^, Open Geospatial Consortium (OGC)^19^, and openEO^20^ has enabled the development of API tools for accessing and processing geospatial data. Additionally, integrated solutions such as the Infrastructure as Service (IaaS), Single-Sign-On (SSO) and S3-compatible data storage access^21^, now support the efficient operation of large-scale cloud processing systems for EO data.

Solving the challenges of data volume, access, processing and sharing individually is possible, but these developments enable solving them in an integrated way. This has potential to create a transformative solution, fundamentally changing the way EO data is managed, shared and used. However, establishing and operating such a platform at the necessary scale demands independence from individual market interests in the form of a public investment. As stated in the Compendium of EO Contributions to the SDG Targets and Indicators^22^, “The main limitation now is not if EO data exists but where it can be stored, accessed and in a format ready to be used”.

Focusing on the challenges, Copernicus Data Space Ecosystem (CDSE), was created to provide an integrated set of data access and computing tools, facilitating the processes that result from downloading, storing and local-side processing of the multi-petabyte EO data. CDSE fills the gap by enabling access to most of Copernicus satellite data and supporting EO missions via a public, centralised platform with generic data access protocols. The ecosystem aims to provide a comprehensive set of services that lower the bar for newcomer EO users and developers while offering a high-performance infrastructure for advanced users backed by a long-term public-funding commitment and governmental governance. It helps governments make informed decisions and enables industry to integrate EO imagery from CDSE into commercial products. CDSE and Data supply are governed by the Copernicus Programme evolution, as established with the entry into force of the “Contribution Agreement between the European Commission, representing the European Union, and the European Space Agency on the Implementation of the Union Space Programme and Horizon Europe”. CDSE service level performance is made transparent to the public through a daily updated dashboard. Services are reviewed by and discussed with the global user community on a regular basis by means of surveys and user review meetings and the resulting roadmap is continuously updated on the web portal. All data and services are located and managed in EU Member States only and comply with the relevant ISO security standards. The terms and privacy policy ensure all personal data and IPR are protected according to EU regulations.

This paper provides the scientific community with a comprehensive, authoritative, and citable technical description of the CDSE platform, thus filling the gap in the existing literature for a systematic introduction to this emerging critical infrastructure.

## Data and Methods

**Fig. 1:**
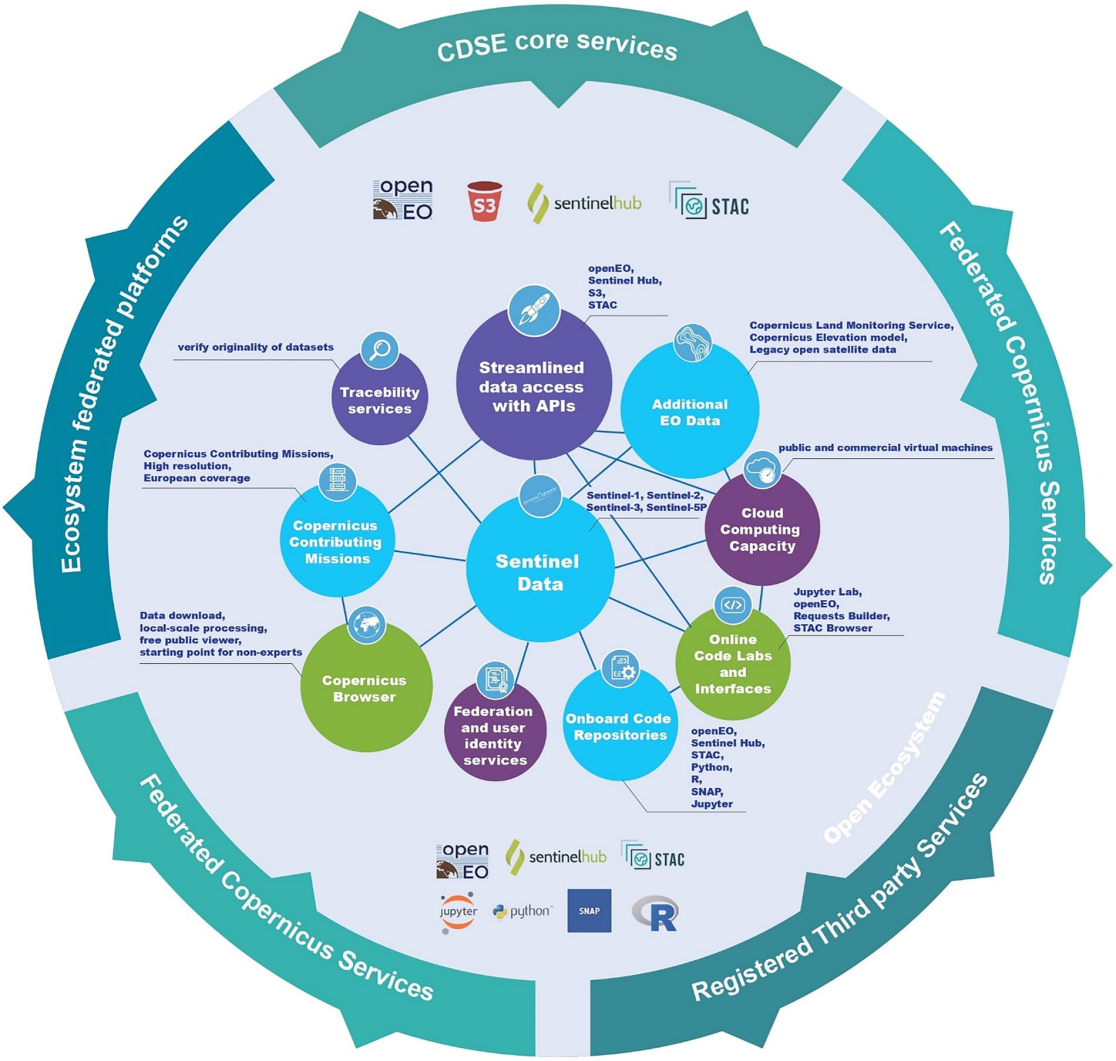
Overview of CDSE datasets, tools and capabilities.

CDSE provides access to all Sentinel data, while also offering Contributing Mission and complementary data to support downstream applications as shown in Supplementary material Table 1. Processing methodologies (Fig. 1) provided by CDSE are described in the second part of this section.

### Data collections

#### Sentinel-1

Sentinel-1 (S-1) is a three-satellite, polar, sun synchronous constellation providing synthetic aperture radar (SAR) measurements with a revisit cycle of 6 days over Europe. The data is currently collected according to an observation scenario covering most populated regions of the Earth. S-1 products are particularly suitable for emergency response, marine surveillance, ice monitoring and interferometric applications such as detection of subsidence and landslides^23^. Main applications of S-1 data are land cover classification^24,25^, hydrological monitoring, including soil moisture^26^, and flood mapping^27^ and last but not least vegetation parameter retrieval^28^. Radar imagery is independent of lighting conditions and is hardly influenced by clouds, thus allowing for data collection where and when optical imagery is unavailable. Therefore, polar studies and marine ice mapping also rely heavily on S-1^29^.

The instrument onboard S-1, equipped with an active phased array antenna, supports four imaging modes: Interferometric Wide Swath (IW), Extra Wide Swath (EW), StripMap (SM), and Wave (WV). This imaging C-band SAR instrument (5.405 GHz) acquires dual polarimetric data (HH/HV or VV/VH). The spatial resolution depends on the swath mode, at IW mode the swath is 250 km with 5 m × 20 m footprints, whereas, at EW mode the swath is 400 km with 20 m × 40 m ground resolution.

CDSE only serves S-1 data collected over land, coastal areas and inland seas (such as the Baltic and the Mediterranean). S-1 data provided is at Level-1, with radiometric correction and terrain correction applied. Optionally, uncorrected data is also available. Furthermore, S-1 Level-3 mosaics have recently been introduced. The mosaicking processing chain involves correcting the slope effect by local resolution weighing the global composition of Level-1 dual polarised products by^30^.

#### Sentinel-2

Sentinel-2 (S-2) provides multi-spectral high-resolution optical observations over global terrestrial surfaces with high revisit times^31^. Consequently, S-2 images are the basis for land-cover and change detection maps^32,33^, various bio-geophysical variable maps^34^, contributing to land monitoring, emergency response and security services. The spectral characteristics of S-2 are sensitive to crop parameters, thus, one of the most prominent applications of S-2 data is agriculture monitoring in the framework of the EU Common Agricultural Policy (CAP), EU Deforestation Regulation and precision agriculture^35–37^. S-2 also plays a role in sustainable management of inland waters and coastal areas, with successful large-scale application for bathymetry estimation^38^. The short-wave infrared bands of S-2 are widely used for monitoring active fires and other thermal hot spots and burn scars in the landscape^39^).

S-2 is placed on a sun synchronous orbit at an altitude of 786 km, with 5 days of revisit time at Equator (with S-2A, B and D platforms in orbit)^40^. The Multi-Spectral Instrument onboard S-2 features 13 spectral bands in the visible (VIS), near infrared (NIR) and short-wave infrared (SWIR) regions. The spatial resolution ranges from 10 m to 60 m depending on the wavelength, with a swath width of 290 km.

Within the CDSE environment, S-2 data is served as Level-1B imagery without atmospheric correction, Level-2A with atmospheric correction, cloud masking and basic surface classification applied using the standard Sen2Cor algorithm, and Level-3 data in the form of Quarterly Mosaics that are generated as a composite of the cloudless pixels^41,42^.

#### Sentinel-3

The Sentinel-3 (S-3) mission was specifically designed to ensure a long-term collection and operational delivery of measurements of ocean, land, and atmospheric properties, while contributing to emergency and security services^43^. With S-3A launched in February 2016, the mission aims include the continuation of its predecessor ENVISAT. Its primary objectives are optical measurements from ocean, inland sea and coastal zones and Sea/Land surface temperature and topography estimates. Amongst many, geo-biophysical products obtained from S-3 imagery include sea^44^ and land surface temperature^45^, leaf and canopy variables^46,47^ as well as sea surface height estimates^48^.

S-3 is also a system of two satellites, each carrying a set of instruments optimized for monitoring marine and land parameters at global scales. These include the Ocean Land Colour Instrument (OLCI), a wide swath, high spectral resolution imager working with 21 spectral bands in the 390 nm to 1040 nm range. OLCI has a spatial resolution of 300 m and provides 2-3 days of global coverage revisit times.

The Sea-Land Surface Temperature radiometer (SLSTR) is a conical scanning imaging radiometer that supports robust atmospheric correction and accurate sea-land surface temperature retrievals, from its channels in the VIS, thermal infrared (TIR) and SWIR spectrum^49^. SLSTR has 500 m spatial resolution at visible wavelengths and 1 km spatial resolution at shortwave infra-red to infra-red wavelengths. The revisit time is approximately 24 hours^43^.

The SAR Radar Altimeter (SRAL) onboard S-3 is a dual-frequency Ku and C-band nadir-looking radar altimeter. The instrument offers 300 m along-track measurements, optimizing data collection over diverse terrain such as coastal regions, sea-ice boundaries, and inland water areas. This mode enhances information retrieval in challenging environments that conventional pulse-limited altimeter systems find difficult to measure^50^.

S-3 OLCI, SLSTR and SRAL data are served as separate collections on CDSE. OLCI imagery is served as Level-1B, and recently also Level-2A, with atmospheric and radiometric correction. SLSTR data are served as raw band values and brightness temperatures. Measurements from OLCI and SLSTR are combined to generate Aerosol Optical Thickness and Surface Directional Reflectances for 300 m pixels in S-3 SYNERGY Level-2 products. SRAL data is served as Level-1 and Level-2 products, which already include geophysical products such as Sea surface height anomaly, significant wave height and wind speed.

#### Sentinel-5P

Sentinel-5P (S-5P) is a low Earth orbit polar satellite dedicated to assessing the air quality, climate and the ozone layer^51^. Onboard S-5P is the Tropospheric Monitoring Instrument (TROPOMI), an imaging spectrometer measuring key atmospheric constituents such as sulphur dioxide (SO_2_), ozone (O_3_), carbon monoxide (CO), methane (CH_4_), nitrogen dioxide (NO_4_) and formaldehyde (CH_2_O). Launched in 2015 with a single-satellite single-payload system, TROPOMI aims to better constrain the spatiotemporal variability of the trace gas sources, improve upon the understanding of climate forcing by aerosols and to provide improved input for global air quality and climate models^52^. Apart from various atmo-tropospheric constituents, applications of TROPOMI based data include the estimation of solar induced chlorophyll fluorescence and volcanic activity monitoring^53–56^. In order to track rapidly varying conditions in the troposphere, TROPOMI combines daily, global coverage with a spatial resolution of 7 × 7 km^2^, with a wide sensor swath of 2600 km.

The TROPOMI instrument is a push-broom sensor with spectral bands in ultraviolet (UV) and visible from 270 to 500 nm, the near-infrared (NIR) from 675 to 775 nm and the shortwave-infrared (SWIR) band from 2305 to 2385 nm. The spectral resolution ranges from 1 nm in the shortest UV band, to 0.25 nm in the SWIR, and 0.5 nm in the other bands.

Sentinel-5P is served in CDSE as data products directly representing the concentration of various pollutants, as L2 data. The L2 catalogue consists of datasets describing the total columns of ozone, sulphur dioxide, nitrogen dioxide, carbon monoxide, formaldehyde, tropospheric columns of ozone, vertical profiles of ozone and cloud & aerosol information.

#### Copernicus services

Copernicus Services are based on data collected from Earth observation satellites and *in situ* sensors’ observations. Within the CDSE, users can find global datasets from the Copernicus Land, Atmospheric, Marine Environment Monitoring Services, and Emergency Management Service. Global datasets from Copernicus Land Monitoring Services are provided via the Sentinel Hub APIs and Copernicus Browser, data from the Copernicus Emergency Management and Copernicus Atmospheric Monitoring Services are currently served through S3 access using REST APIs.

Copernicus Services provide both near real-time and historical data. The wide range of biophysical variables and reference data from local to global scale supports numerous applications, including agriculture and food security monitoring, weather forecasting, climate change impact assessments, disaster management and the assessment of atmospheric composition^57–59^.

Among these services, currently the Copernicus Land Monitoring Service (CLMS) is best integrated into CDSE – currently nearly all global datasets are available via S3, OData, the Sentinel Hub APIs and Copernicus Browser. CLMS features six main thematic areas (1) Land Cover and Land Use Mapping provides detailed land cover classifications at pan-European and global scales. Pan-European data include layers on features like forests, grasslands, and water, while global mapping aligns with the FAO’s Land Cover Classification System^60^. (2) Priority Area Monitoring focuses on delivering customized and detailed land cover and land use information for specific areas of interest, often referred to as “hot spots”^61^. (3) S1 and S2 Global cloudless mosaics are also an integral part of CLMS catalogue. (4) The systematic monitoring of Bio-geophysical Parameters on land surface status and evolution provides data on soil moisture, snow, vegetation, temperature, surface reflectance, and water bodies, now directly available as data layers in CDSE^62^, and ultimately^6^ Reference and Validation Datasets are also available (through the CLMS portal, not CDSE) for validating core biophysical products.

The Copernicus Atmosphere Monitoring Service (CAMS), operated by the European Centre for Medium-Range Weather Forecasts (ECMWF) provides regular data on atmospheric composition. Some selected CAMS datasets are available from the CDSE eodata catalogue. CAMS provides real-time analyses, forecasts, reanalysis, and retrospective records of atmospheric conditions exploiting over 90 different satellite data streams. The CDSE supplies data from the Global Fire Assimilation System (GFAS), Global Atmospheric Composition Forecasts with vertical-level analysis and forecasts, Global Additional and World Meteorological Organization (WMO) Essential datasets, including cyclone-related information^63^.

The Copernicus Marine Environment Monitoring Service (CMEMS) provides insights into the physical (blue), sea ice (white), and biogeochemical (green) dynamics and climatological variations of the global oceans and European seas, integrating Sentinel imagery and reference data^64^. The CMEMS datasets within CDSE have a global extent employing both optical and radar imaging techniques. These datasets are planned to be shared in CDSE by directly mirroring the CMEMS STAC Catalog.

The Copernicus Emergency Management Service (CEMS) delivers geospatial data and imagery for decision support related to natural or human-induced disasters. CEMS continuously monitors the planet for signs of impending disasters or real-time occurrences. One key offering from CEMS is Rapid Mapping, providing geospatial information within a service request to support emergency response efforts in the immediate aftermath of a disaster. Rapid Mapping datasets are organized by the type of emergency, such as floods, wildfires, earthquakes, epidemics, humanitarian crises, industrial accidents, landslides, storms, or volcanic activities^65^.

#### Federated, complementary and contributing missions datasets

In addition to the complete online archive of Copernicus missions, additional datasets are continuously integrated over time through a federated approach. This expands the data offering and brings the benefit of using complementary data for enhanced analytics, e.g. high-resolution optical images and digital elevation models. The selection of such datasets is gradually extended and is planned to cover the full portfolio of the Copernicus services in the future. These Copernicus Contributing Missions (CCMs) are operated by European and international third-party organizations, supporting the needs of Copernicus Service Providers. CCM missions are an essential component of the Copernicus programme, which help meet the requirements of Copernicus Services and other users. A major source for auxiliary data comes from the Copernicus Contributing Missions (CCM). The most recent SAR CCMs include the COSMO-SkyMed, PAZ and ICEYE, whilst numerous missions collect information from the optical domain, including Pleiades Neo, HiVE, and BALKAN-1. CCMs are available in the form of three mosaics with near-complete European coverage, from 2018, 2021 and 2024.

Additional datasets from third parties, such as S-1 Radiometric Terrain Corrected backscatter and Orbits, as well as Sentinel-2 global mosaics and land cover (Europe and Poland), and Digital Elevation Models (COP DEM^66^ and SRTM DEM (Yang, 2011)) are available. Furthermore, complementary federated datasets such as PROBA-V or S-5P Level 3 products are accessible via the openEO federation endpoints, complemented by the Terrascope platform, which is the first of its kind to truly integrate into the CDSE openEO federation. Terrascope serves as Belgian’s national EO ground segment, serving regional and global added value products and services towards their community. In addition, public datasets beyond Copernicus are also partly integrated, including the widely used Landsat-5,7 and 8 missions, standing as the world’s longest-running satellite system dedicated to moderate-resolution optical remote sensing developed by the National Aeronautical and Space Administration (NASA) and United States Geological Survey (USGS)^67^. Moreover, imagery from ENVISAT-MERIS is also available, allowing for long-term, multi-decadal scientific investigations^68^. These datasets are privately governed but provide local processing capacity next to the data to be able to have a multi-back-end setup for users, increasing open-science capabilities. Auxiliary datasets collected during satellite operation with onboard GNSS and attitude sensors and from system telemetry are also available to users. User-defined pre-processing pipelines rely on these datasets, and additionally, they are also used for anomaly monitoring. The datasets are available in their raw formats, according to the satellite, sensor, area and time of interest.

### Processing methodologies and service technical solutions

**Fig. 2:**
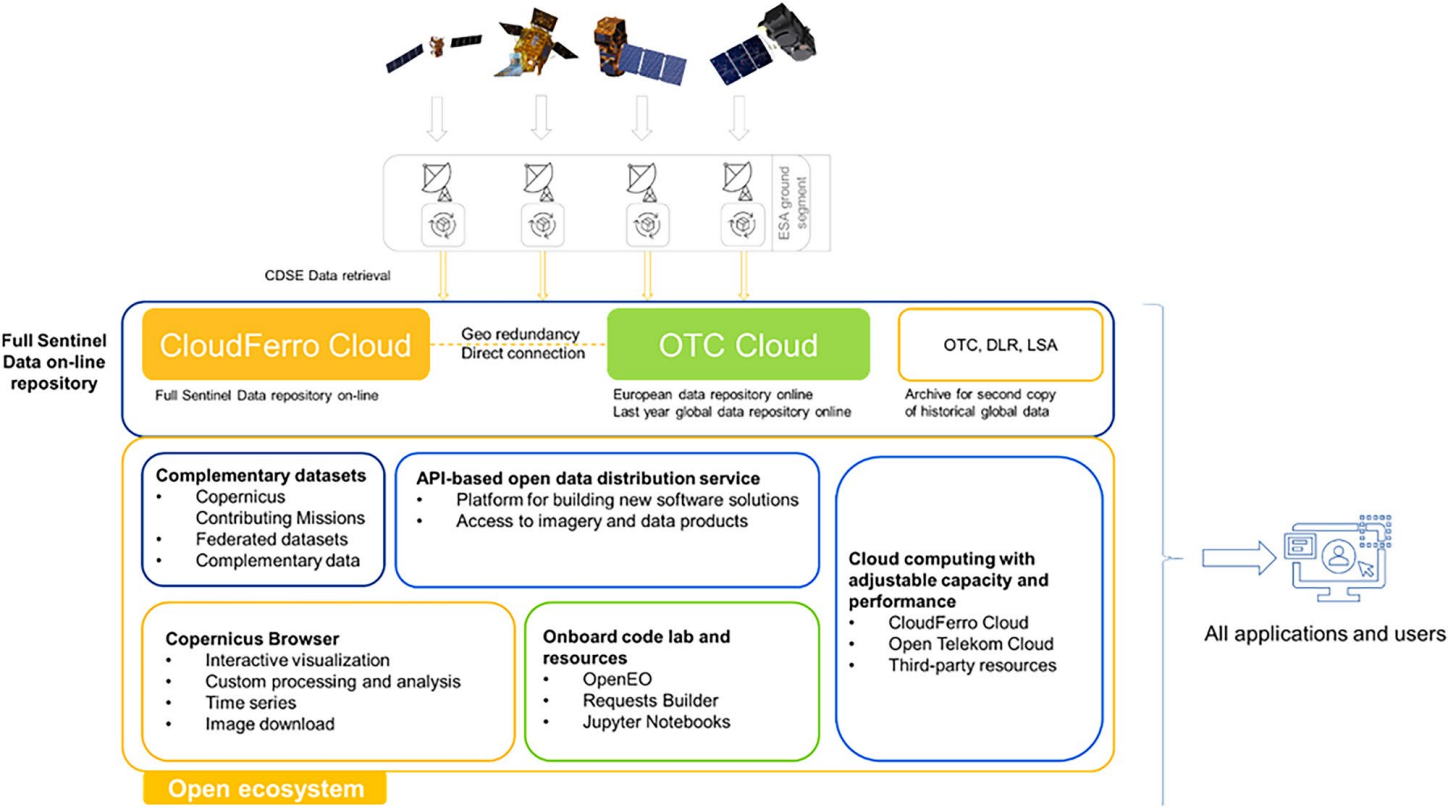
General architecture of CDSE.

The CDSE platform runs on top of two Public Clouds, CloudFerro Cloud (CFC) and Open Telekom Cloud (OTC). The clouds are directly connected to each other, the GÉANT scientific network, and the internet, using redundant broadband, low latency multiple 100 G links to allow smooth operation across the clouds. The applications and components composing the platform are installed in virtual environments using standard cloud resources such as Virtual Machines, Virtual Networks, Containers, Block and Object Storage, on both clouds. The EO data are stored on the CFC and OTC and additionally secured in the OTC as well as in DLR and LSA archive for backup (Fig. 2). The store policy is designed to maximise the system security, meaning that each dataset has at least two copies in different georedundant locations.

While the data and services are available through the web portal and GUI, the API services are the core of cloud computing on CDSE – by providing a machine-to-machine interface, they allow users to perform individual processing operations or build operational systems using on-demand processing as part of the data request. API access is not entirely new: already the Copernicus Open Science Hub supported automatic, API based downloading of images from the repository. However, CDSE is optimized for API-based cloud processing, opening new functionality compared to the Open Science Hub through the integration of APIs that support analysis as part of the API request. A visual overview of the CDSE architecture can be seen in Fig. 2.

#### The Catalogue - OData, STAC, OpenSearch APIs

CDSE is compatible with established APIs of the EO community, that offer direct download functionality for user-defined areas and time frames of interest. The data can be searched and accessed with three main catalog API interfaces.

OData is an ISO/IEC approved, OASIS standard^69^, which is based on https RESTful Application Programming Interfaces. OData makes it possible to build REST-based data services that let Web clients publish and edit resources that are recognized by Uniform Resource Locators (URLs) and described in a data model using straightforward HTTPS messages.

STAC (SpatioTemporal Asset Catalog) is a relatively new web service specification for catalogues, that is increasingly used and supported^18^. STAC data have become a de-facto standard in the EO community, also being onboarded to OGC. STAC items are provided for all online products, as well as for products generated by users within the CDSE. It is worth noting that as the STAC API is currently actively developed by the community, new versions are being introduced to the CDSE as they are released. The list of APIs and their corresponding available datasets is shown in Supplementary material in Table 1.

**Table 1:** Benchmark results of openEO data providers.

Data Provider	Total # processes	Latency (ms/KB)	Submission time (s)	Processing time (s)	Download time (s)	Total time (s)
CDSE	138	0.64 ± 0.12	0.67 ± 0.60	204.24 ± 72.57	9.94 ± 1.22	326.47 ± 103.75
EO4EU	97	3.93 ± 1.34	—	—	—	—
EODC	152	1.16 ± 0.27	—	—	—	—
EURAC	78	3.89 ± 0.52	—	—	—	—
GEE	97	1.41 ± 0.17	*0.16 ± 0.00	*10.69 ± 5.10	*0.34 ± 0.08	*11.56 ± 5.07
mundialis	77	1.76 ± 0.33	—	—	—	—
openEO Platform	167	0.74 ± 0.46	3.39 ± 6.40	210.40 ± 21.60	6.60 ± 8.89	308.18 ± 52.48
Sentinel Hub	98	1.04 ± 0.06	—	—	—	—
VITO	141	0.24 ± 0.06	0.65 ± 0.15	194.72 ± 26.78	3.52 ± 0.71	281.08 ± 28.78

Results refer to a process from mean reduction along the temporal domain of stacked pixels, 10 m Sentinel-2A used over three months of data (01/04 – 30/06 2024), spatial subset of 80 km^2^. These results refer to the study by^79^. The “*” symbol indicates the coarse resolution (100 m) of GEE.

#### Sentinel Hub APIs

Sentinel Hub API family is a set of RESTful APIs designed for accessing satellite imagery. However, instead of merely subsetting the data by space, time, quality or spectral bands, Sentinel Hub supports quite complex analysis operations as part of the API request. This is enabled by the “evalscript”, a piece of JavaScript code that is part of the request. Evalscripts support any mathematical operation that can be coded using JavaScript language, with three key parts: The setup function defines the input band datasets and algorithm outputs per pixel, the “evaluatepixel” function defines pixelwise mathematical operations on these inputs, and the “evalscript” ends with a list of the final output products, including both quantitative results and RGB values for visualization. This has been shown to enable the calculation of spectral index operations, also pre-defined regressions and classifications, decision trees, and even the fusion of data from different sensors.

Sentinel Hub APIs include the Catalog API, returning a list of image files filtered by the request, the Processing API, returning a raster image created by an evalscript algorithm, and the Statistical API, which integrates an area of interest (as a vector boundary), and calculates statistical products of the pixel values output by the evalscript. Additionally, the Sentinel Hub OGC API supports the creating of web map product services (e.g., WMS, WMTS) from the output of an evalscript operation. These can be loaded to GIS software or web mapping applications – or directly visualized through the Sentinel Hub QGIS Plugin. These APIs also have their Batch counterparts which are optimized for large-scale asynchronous processing. Batch Catalog, Batch Process and Batch Statistical API can run several instances of parallel processing in an optimized way, pushing the results to object storage. Additionally, the Sentinel Hub API family also supports the integration of datasets contributed by the user. These can be converted to the Cloud-Optimized Geotiff (COG) format using standard tools such as GDAL, made available in the CDSE cloud using an S3 bucket and ingested with the Bring Your Own COG (BYOC) API to create datasets that are also compatible with the Sentinel Hub APIs. Therefore, CDSE also enables publishing datasets generated by third parties, enabling not just download access but online processing and even visualization in Copernicus Browser.

#### Copernicus browser

Natural disasters and rapid global change and misinformation create a demand for visualizing processes that normally happen beyond scales people can easily observe. Satellite imagery is a particularly powerful tool for this, providing information across scales of decades and thousands of kilometres. Meanwhile, as EO literacy is growing, more people grasp an interpretation of satellite imagery and their scientific significance. This creates demand for a satellite image viewer for the public which is easy to operate but can convey quantitative information.

**Fig. 3:**
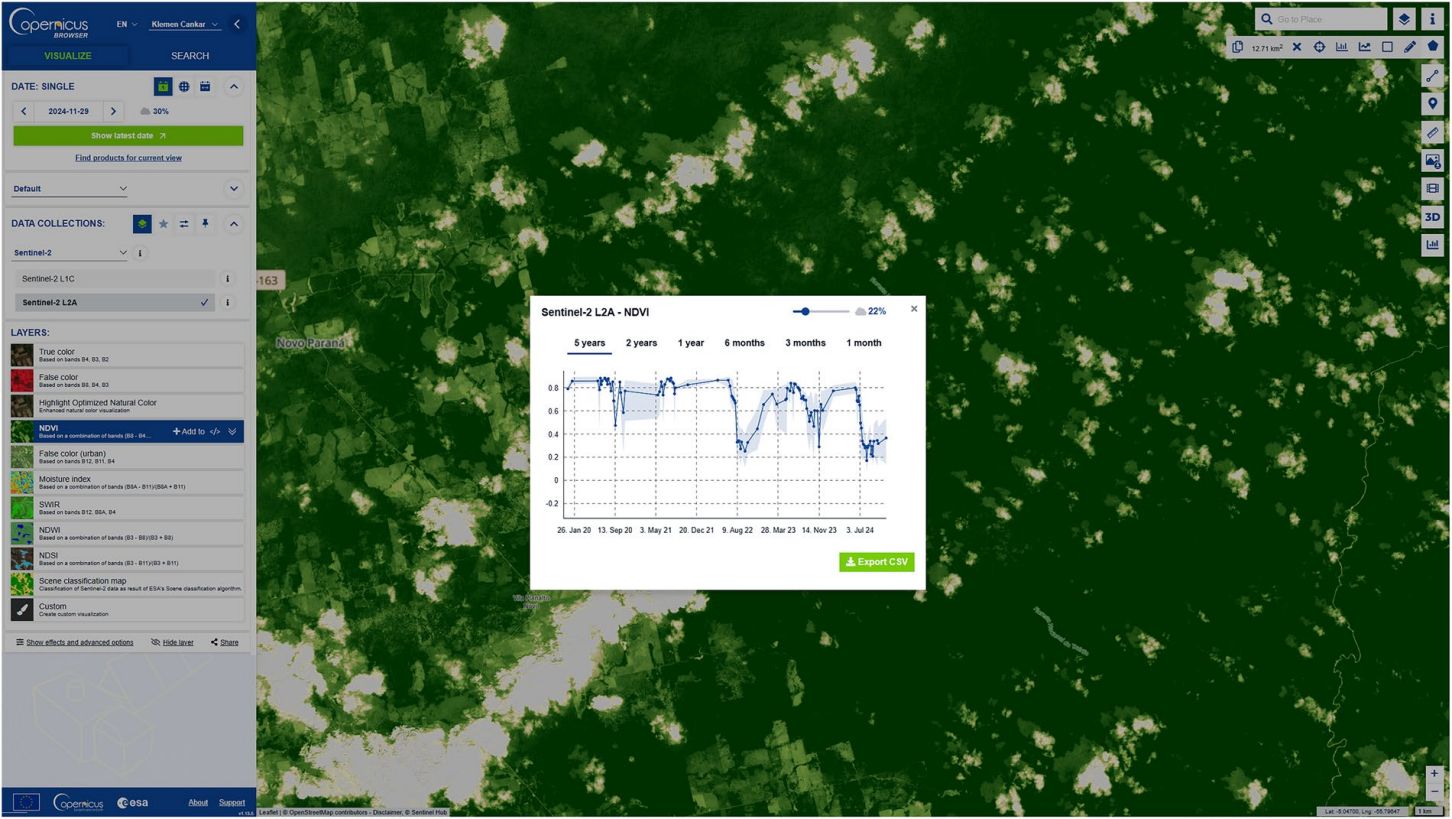
Example of the Copernicus Browser interface, showing an area affected by deforestation near the Xingu River in Brazil.

In Fig. 3 the CDSE Copernicus Browser is depicted, an interactive online visualization, download, and analysis tool. Its objective is to provide easy access to large-scale EO data on the web, with minimal EO specific programming or GIS. The Browser enables viewing, panning and zooming into the selected EO dataset, with filtering and mosaicking based on data quality and the area of interest. To support change detection, dynamic scene comparison can be generated by overlaying multiple raster layers, and time series can be exported in various formats ranging from comma separated values to animated imagery. For scripts that generate a numerical, single-band output, the Browser also supports statistical operations such as calculation of histograms or time series within an area of interest. Individual scenes can be shared with direct hyperlinks and can also be downloaded at user-defined spatial resolutions. Copernicus Browser is also a tool for downloading individual satellite granules and tiles. A dedicated search tab enables detailed queries, imagery can be previewed and downloaded directly or earmarked for bulk download through the CDSE data workspace, which is an integrated platform for asynchronous processing tasks involving large data (e.g., generating SAR coherence). The Browser also supports user-defined configurations. These are combinations of a location, a dataset and a set of layers with their own evalscript that can be saved, shared with other users and opened directly. This way, simple monitoring systems can be directly based on a processing pipeline defined as an evalscript and a custom configuration. This functionality is based on the CSDE backend and the Sentinel Hub APIs.

#### Codebases and coding platforms

EO code development is a complex task that requires a wide skillset. A wide range of coding languages, libraries, modules and platforms makes choices difficult and maintaining a coding environment time consuming. Also, since these are typically complex operations involving large datasets, prototyping and testing is tedious unless carried out on a very powerful hardware platform. As a solution, CDSE offers a Jupyter Lab virtual code development environment and graphical interfaces (openEO, Requests Builder). Jupyter^70^ is rapidly gaining popularity in many fields of scientific and industry computing. The combination of code, formatted text and interactive graphics is particularly attractive in an EO setting where the outcome of processing operations can be instantly explored. Jupyter also supports sharing of knowledge, since processing pipelines defined in notebooks are easy to adapt and test in new locations and settings. Jupyter can act as an integration framework, calling libraries in various programming languages. CDSE Jupyter Lab offers an interactive cloud-based coding environment: When the user opens the lab, they can launch a dedicated virtual server with the CDSE data collections already on board and a selection of kernels with various languages and libraries (Julia, Python, R). A repository of code examples with Sentinel Hub and openEO APIs supports hands-on learning step by step. Jupyter Lab is not just a tool for education, but for experimenting, prototyping and running local analyses in a professional setting.

#### openEO

Accessing different cloud computing platforms currently require the execution of distinct, platform-specific communication strategies as each platform architecture is designed uniquely. The openEO API standardizes EO interactions between local clients (R, Python, and JavaScript) and cloud service providers, facilitating data access, processing, and comparability. By enabling standardized communication and concealing architectural differences between cloud platforms, openEO facilitates compatibility^71^. The API translated standardized commands interact with EO data and metadata from the backend as virtual data cubes. This approach incorporates key data cube principles and aligns with the emerging standards of STAC involving the typical data cube aspects, as described in^72^ and^73^. Operations with data cubes are represented by a process graph which can store sequences of pre-defined operations and can also be edited in a graphical user interface.

**Fig. 4:**
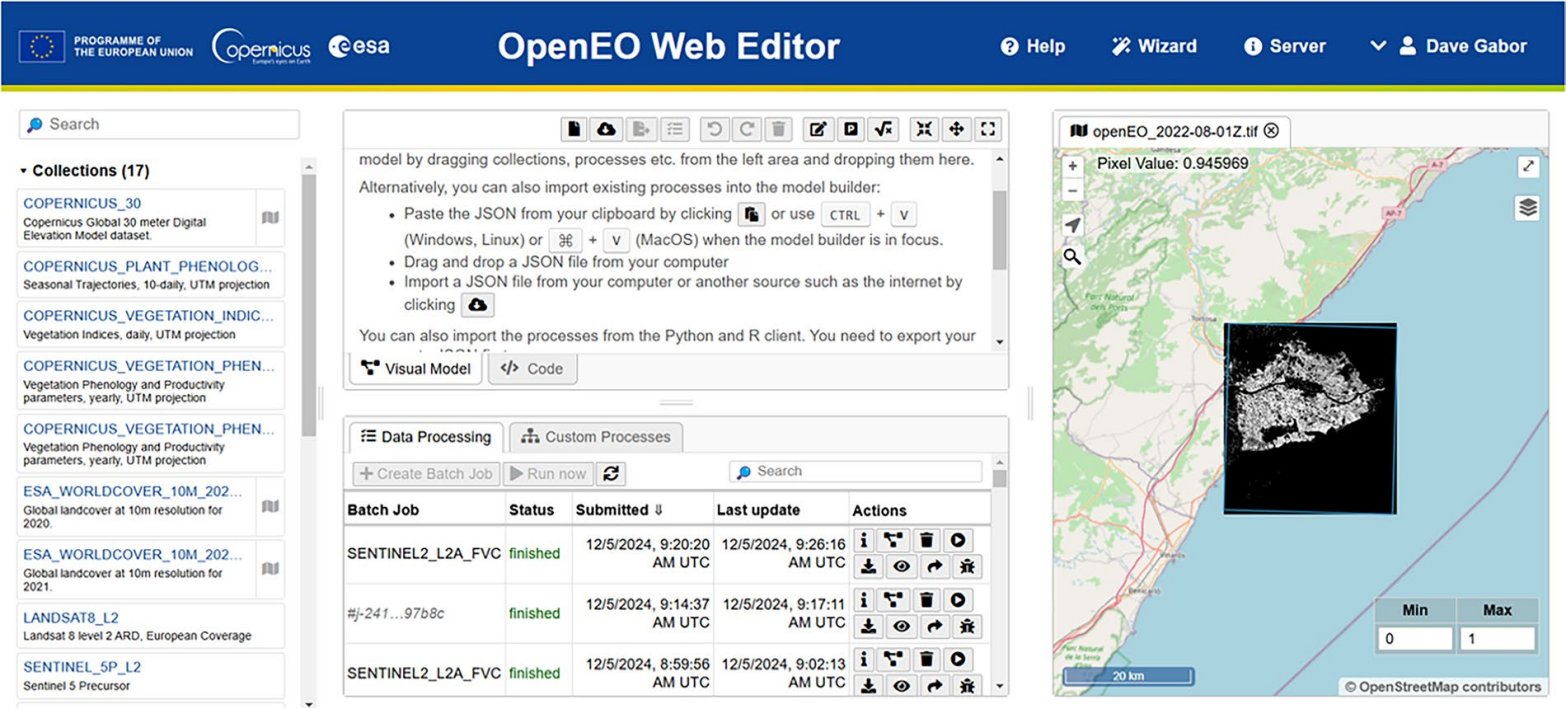
openEO Web Editor graphical user interface. Fractional vegetation cover over the Ebro delta’s rice paddies throughout ripening season, retrieved by Gaussian Process regression algorithms trained on radiative transfer simulations with PyEOGPR^80^ using UDFs.

The API works the following way: first the locally implemented source code is converted to a human and machine-readable JavaScript Object Notation (JSON) that is sent by a HTTP request to the corresponding cloud backend. The process is then executed on the EO data stored on the cloud-back ends. Resulting data can be visualized and downloaded by an additional HTTP request. Such processing workflow enables users to bypass the computing and memory burden imposed by the local processing of EO data^74^. User-Defined Functions (UDFs) are a key functionality of the API. UDFs enable users to upload their custom code for execution that applies a process to each pixel of a scene, allowing for tailored calculations directly on server-side data. The flexibility of UDFs, running on R and Python local clients, allows users to implement virtually any desired functionality, thereby expanding analytical possibilities. This can include bio-physical models, deep learning algorithms, or processing pipelines composed of several individual openEO processes. Since data cubes can be large, platforms may process smaller subsets, invoking UDFs with different data segments to enable parallel execution. Such complex, custom functions are greatly supported by the widely used machine learning (ML) libraries such as TensorFlow^75^ and Keras^76^ or users can develop their own algorithms for numerous use cases. Scientific contribution is fostered by the Algorithm Plaza, where individuals can upload their custom openEO based workflow. The Algorithm Plaza allows for onboarding of third-party services and algorithms, providing developers with an opportunity to expand their reach. Users can run the shared workflows via openEO’s Web editor, which is a graphical user interface (Fig. 4), or use the openEO API to implement them into their programming environment.

The integration of data processing API tools and deep learning models has been significantly advanced within CDSE by the integration of ONNX and openEO. ONNX is a unified format for the representation of deep learning models from various sources (CNN, U-Net, Pytorch). ONNX is a selection of pre-trained models that can be adapted to various solutions. Trained models can be converted to ONNX and run as user-defined functions in openEO, enabling rapid testing, application, and sharing within the Ecosystem. JupyterLab further supports this with user-installed libraries for deep learning development.

#### CREODIAS platform – the commercial component of the CDSE

Communities, public government authorities and industry companies often have to collaborate to deliver EO solutions. Therefore, CDSE has well-defined functionality for open code sharing, data analysis and processing based on quotas for use across participating entities, and commercialization of algorithms for companies. Since user quotas cannot be unlimited, scale-up commercial virtual machine processing capacity is an integral part of CDSE, provided by CREODIAS. CREODIAS features all the EO data collections and functionalities of CDSE in a commercial cloud environment. This enables users to run code that was developed on CDSE with minimal modifications. Furthermore, it provides the opportunity for expansion by enabling 3^rd^ parties to onboard onto the platform and enabling access to paid services such as data hosting, analysing, storing and sharing. It is possible to utilize a geospatial database server for data access, deploy Kubernetes virtual clusters (up to 64 vCPUs and 496 GB of RAM), leverage high-performance GPU-based instances, and employ advanced data access tools such as Open Data Cube.

## Results

### Benchmark tests of processing efficiency

**Fig. 5:**
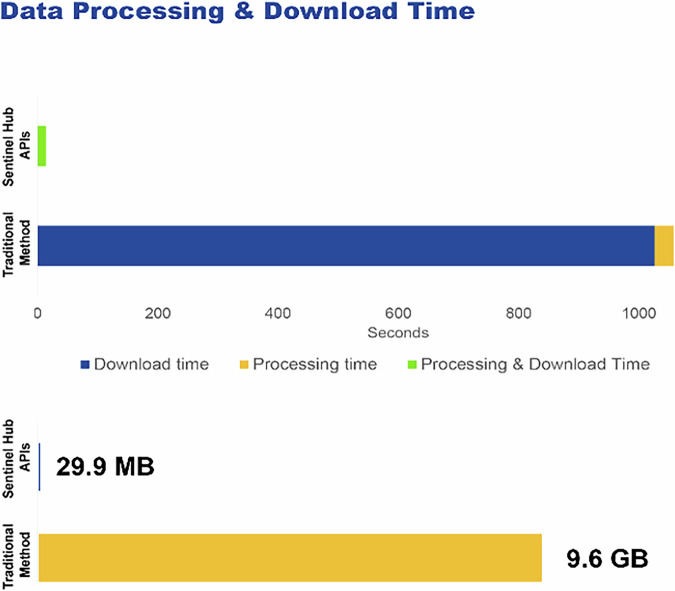
Benchmark test comparing download-based image processing and analysis with Sentinel Hub APIs in CDSE.

Data access is substantially faster and more efficient with CDSE APIs compared to legacy download-based tools. A benchmark study of ^77^, conducted a quantitative assessment of the performance of the traditional, download-based approach with cloud-based API access (Fig. 5). For this case, a basic harvest mapping analysis was carried out for a study area of 193 km^2^ near Paderborn in Germany. The Normalized Difference Vegetation Index (NDVI) and Bare Soil Indices (BSI) indices were calculated, and their changes in a time series evaluated to identify harvested agricultural parcels. The classical approach was to download data in the form of zipped S2 granules, save, unzip, subset and filter, calculate the index on the respective bands, and then evaluate the change of the indices in a time series. This was carried out in Python code as a benchmark, using the OData API (Sharma, 2019) for downloading, Rasterio^78^. for processing the images, scipy for morphological filtering and creating the final product. The CDSE solution involved a Sentinel Hub Statistical API request with an evalscript calculating NDVI, BSI and their changes in the study period. The results show that using CDSE API requests reduces the data volume to transfer from 9.6 GB to 29.9 MB and the processing time from 18 minutes to 5 seconds (Fig. 5).

Another benchmarking study comparing openEO on different back-ends was conducted by^79^. Process availability, latency and processing performance were investigated, on multiple openEO data providers. The number of processes, operations that performs a specific task on a set of parameters and return a result, are the highest on the CDSE, EODC, openEO platform, and VITO providers with more than 130 different functions. Latency test showed the best result on CDSE, openEO, and VITO providers with all less than a millisecond. A reducer process was tested to compute mean pixel values for 10 m S-2A reflectance, over an 8 × 10 km subset (16.318°E–16.425°E, 48.163°N–48.253°N) through three months, April to July 2024. The tested platforms finished around 5 minutes. Note that processing times were nearly identical across all platforms. GEE performed the reduction at a coarser resolution than other platforms, thus resulting in better performance.

**Fig. 6:**
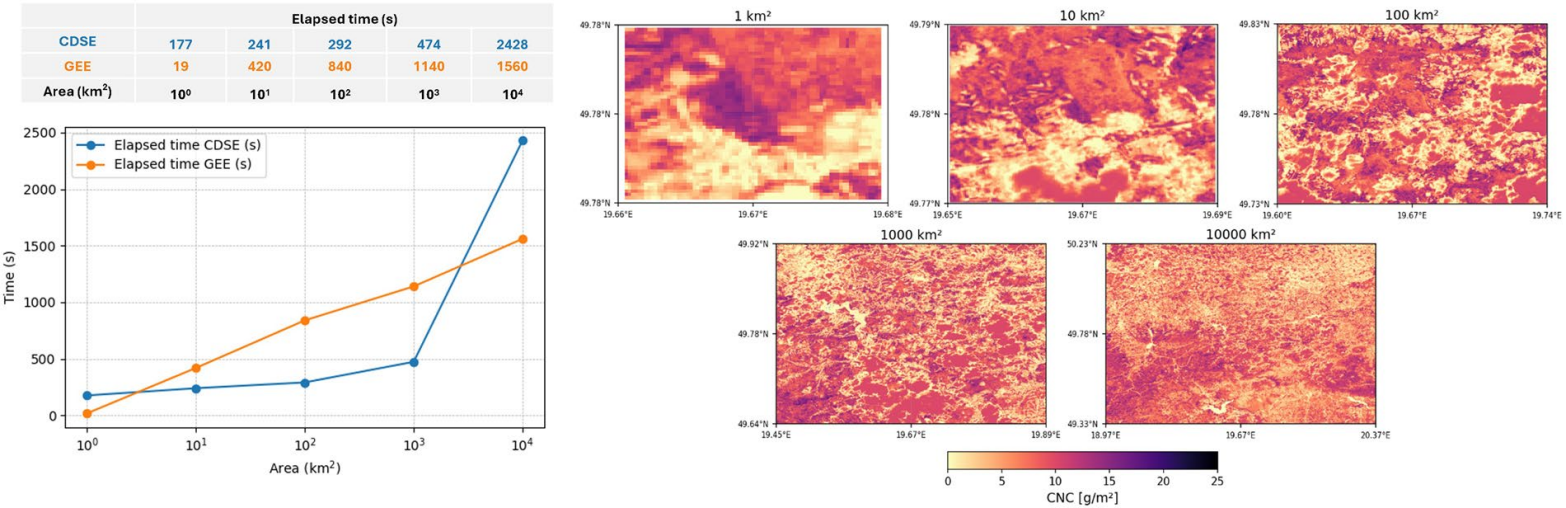
Scaling analysis over Central Europe for 2022 July 1-10th, 10-day temporal composites. Analysis conducted by^80^.

A scaling analysis was conducted by^80^, where a Gaussian Process Regression ML algorithm was used to retrieve 20 m resolution canopy nitrogen content from S-2A data, at incremental areas of interest by a factor of ten. The same algorithm and its runtimes were compared on openEO and GEE. CDSE is notably faster for retrieval, at smaller areas (<10^4^ km^2^). At 1 km^2^ CDSE takes longer, due to possible queueing of tasks in openEO, while openEO was not able to retrieve above 10^5^ km^2^ (Table 1, Fig. 6).

### Paradigm shift towards cloud computing

CDSE enables a new approach to building satellite imagery-based environmental monitoring pipelines. To provide an example of operational use within the environmental monitoring sector, we refer to the Finnish Environmental Research Institute (SYKE) who operate the TARKKA online lake monitoring and modelling application, based on EO data. This system is running on OpenSearch, OData and Sentinel Hub API requests in CDSE, with spectral operations calculated in the cloud and the resulting maps served to users. No imagery is downloaded; images are provided through the Sentinel Hub OGC API to the interface. CDSE Simple Storage Service storage is used. For backwards compatibility of current sensors such as the S-3 OLCI, historic data are also stored on CDSE as Bring your own COG (BYOC) collections. The results are disseminated in the form of online maps and charts to users in an interactive web interface, which includes true colour imagery using the Sentinel Hub OGC API and water quality parameters calculated by the system. TARKKA is used mainly by environmental administrators, but also by citizens tracking ice, water quality, and temperature — about 800 daily users^81^.

Satellite based agricultural monitoring has recently gained significant attention due to the ongoing global food crisis and increasing concerns about food security. Within Europe, the Common Agricultural Policy (CAP) is a key pillar of the continent’s food supply, now requiring national governments to conduct more comprehensive checks and controls on agricultural production and farmers’ payment claims^82^. To address the need for the enhancement and simplification of subsidy compliance monitoring, in 2017 ESA and the EU have initiated the Sentinels for CAP programme (Sen4CAP), that provides validated data and algorithms to agricultural institutions addressing the requirements of CAP. The retrieved EO products include cultivated crop type maps; grassland mowing product, agricultural practices monitoring product, vegetation status indicator, as well as the interactive visualization services for satellite imagery and use-case products^83^, originating from Sentinel-1, Sentinel-2 and Landsat-8. Integrated within the CREODIAS cloud computing platform, these agricultural parameters can be retrieved by the end-user, without the need for local computing resources^84^.

The Food and Agriculture Organization (FAO) defined obstacles to global agricultural mapping, such as lack of availability of long-term products, scarcity of thematic detail, infrequent updates, and the absence of open-source systems for reproducibility and improvement. To tackle these shortcomings, with the necessary tools provided by openEO and CDSE, the ESA WorldCereal project has delivered a global, seasonal, and reproducible crop and irrigation mapping system. The resulting data includes an annual temporary crop map, seasonal maize and cereals map and irrigation maps as well as model confidence layers relating to the individual products. Thanks to openEO and CDSE, these global products can be accurately used for local use cases, by allowing users to fine tune the maps by adding their own training data. From a standalone data product, openEO and CDSE are therefore taking WorldCereal to the level of an open public service, contributing to farming, agriculture monitoring and protection of the environment^85^. Together, projects like Sen4CAP and WorldCereal demonstrate how advanced EO technology and collaborative platforms aid agricultural monitoring, helping global efforts to enhance food security and promote sustainable practices^86^.

### Artificial Intelligence in CDSE

AI tools are transforming the use of large datasets worldwide. Although they have rarely been applied to satellite imaging data, ML methods are widely used, and there is growing demand for closer integration of deep learning tools^87,88^. The main bottleneck is the lack of compatibility between EO datasets, satellite image processing software tools and deep learning libraries. Data integration and the adaptation of processing tools require specific set of skills. CDSE presents advances that hold strong potential for bringing AI-based satellite data processing within reach for startups, university research groups and government agencies.

The recent advances in AI are being adopted by the EO community by the application of different ML algorithms on EO datasets. However, the main limitation towards the application of AI on any dataset is the workload needed for data cleaning and feature preparation^12^. API access to data collections in CDSE is a significant step forward for these initial tasks, since the data are streamed in consistent formats and with consistent quality. Calculating relevant features can be performed in a highly automated and scalable way with the APIs that are also capable of asynchronous processing. Additionally, S-1 and S-2 datasets are now being provided as Level-3 Analysis Ready Data. This means that even for large study areas, seamless, effectively noise-free datasets are available. This will practically eliminate the cumbersome task of data preparation for AI processing. The risk of “hallucination” - i.e. the model falsely learning features that do not represent the actual information in the dataset is also considerably reduced in case Level-3 Analysis Ready data mosaics are applied.

At a further level, it is now possible to serve Sentinel data in the form of “embeddings”. Embeddings are high-dimensional vectors that transform complex data into numerical representations. By providing an optimum reduction of the data while capturing relationships and semantic meanings, embeddings make datasets available for AI models while supporting context awareness. Embeddings support the application of foundation models (powerful AI systems pre-trained on very large datasets), thereby enabling object detection or image classification with high performance. Embeddings prepare data for similarity search, quickly identifying similar locations and features across the globe. Also, embeddings developed in Major TOM standard developed by ESA can be integrated, enabling faster and more accurate inference and fine-tuning of AI models with satellite data. CloudFerro and ESA Phi-lab have recently released the first global scale EO embeddings that are available on CREODIAS. Based on this first successful test, additional embeddings will be generated for other data sources and time steps and provided within the CDSE.

## Discussion

### Comparison with other EO cloud platforms

CDSE encloses more diverse data access, processing and visualization solutions (e.g.: Copernicus Browser, openEO, APIs) when compared to other platforms e.g., Google Earth Engine (GEE) and Microsoft Planetary Computer (MPC). GEE, MPC and other alternatives are all similar that they feature a visualization interface, however, e.g., MPC can only show raw satellite granules, without the possibility of pixelwise algorithmic interaction. Most platforms are more designed for data access, rather than scientific algorithm development. Both CDSE and GEE allow for calculations via an evalscript-like method, to visualize user-processed EO products, such as spectral indices. Both these platforms feature a high level of abstraction, clearly showing users how and where data is processed, but with restricted use of individual pixel approaches^89^. Whilst these platforms show basic processed results, for more complex processing, users have to use the GEE Python API and openEO or Sentinel Hub in case of CDSE. APIs interact with the data stored on either CDSE or GEE, but users can use their developer interface as well as extend the analysis with programming libraries not available on the platforms’ visualization interface. The benchmark tests conducted by^80^ provided a clear comparison on how openEO on CDSE and GEE perform, while running ML models, indicating that CDSE proved to be faster at smaller scales, while GEE is faster at larger scale retrieval. A limitation for openEO on CDSE was its inability to work at spatial scales of 10^5^km^2^ for 20 m resolution pixels, showing GEE’s superiority for larger scale processing. When comparing the codebases of GEE and MPC, it can be observed, that processes, algorithms and functionalities are not transparently shared with the public, ultimately leading to possible difficulties throughout development. Furthermore, the GEE and MPC the terms of use limit the protection of developer’s intellectual property (IPR). On the other hand, openEO´s codebase is fully open, enabling for a better debugging experience^90^ and ensure IPR protection.

### Challenges and limitations

CDSE faces a common challenge: users stick to established tools, slowing adoption of new ones. While new services like openEO and the Batch Statistical API are emerging, legacy options such as STAC and OData for downloading imagery remain dominant. Transition is ongoing, but legacy solutions are still widely used. Using the Technology Acceptance Model (TAM)^91^ have concluded that the disadvantage users see in the adoption of cloud computing is the lack of full control over the hardware, explaining the slower shift towards CDSE’s cloud-based processing. This effect can be also noted within the context of Sen4CAP, which initiated six years before CDSE, and many agencies show reluctance to change already functioning and legally certified processing pipelines^92^.

As the initial phase of onboarding all main data collections (e.g.: Sentinels, Copernicus Services) and API tools are nearing completion, the next phase is expected to be the evolution of data federation. A unified data repository containing all EO and geospatial datasets is unlikely to exist. However, if various data spaces, repositories, and cloud platforms adopt standardized verifiable credentials and adhere to common data standards, users can develop tools capable of integrating data from multiple sources. The openEO API is already capable of ingesting any STAC-compliant dataset and facilitates this integration to some external platforms complementing the native set of CDSE data collections extent^90^. With Open Telekom Cloud providing an AI stack for large-scale crop monitoring and CREODIAS offering deep learning–optimized VMs, the next step is for major meteorological, ecological, and geoscience repositories to adopt CDSE as an identity provider. Progress is slow, but it could open new opportunities for cross-disciplinary research and operational solutions.

### Opportunities and Future outlook

One key benefit of CDSE is that it is one of the most adopted platforms with more than 500,000 registered users and thus greatly accelerates the exchange of data, code and knowledge. The expansion of the community of researchers and developers enabled by seamless access to a wide range of data analysis services leveraging cloud and high-performance computing platforms advance knowledge creation and foster the development of open-source tools that benefit the broader community. The combination of API requests and the Browser provide a clear pathway for novice users towards learning professional EO code development and potentially becoming service provicers. This integration enables fast prototyping and testing of new methods or products, while allowing their global, space- and time-explicit evaluation. Therefore, research and product development projects in the environmental science domain are increasingly relying on CDSE^93^. Most services, applications and APIs by CDSE can be handled with Python, catering for the requirements of numerous scientific communities. The recent advent of Julia, R and JavaScript clients to program with openEO and Jupyter, provides further options to additional user groups. Future implementation of APIs and various programming languages will facilitate the onboarding of newcomers to the platform.

To enhance the dynamics of shifting users onto cloud computing, one principal objective of CDSE is to educate people from all communities, by providing (multilingual) webinars, hackathons^94^, user review meetings and supporting Massive Open Online Courses^95^. Additionally, User Uptake initiatives have been employed to increase awareness, dissemination, and competencies, thus supporting the development of downstream applications^96^. Throughout education at all levels, interactive digital tools, for example the Copernicus Browser, are becoming increasingly popular, with the growing need to implement remote sensing and geodesy into the curriculum^97,98^.

EO programs are vital to climate change adaptation and mitigation efforts, forming a core component of many business solutions^99^, and selecting a computing platform to utilize EO data is a critical decision with significant financial implications. The offering of CDSE seems particularly compelling for startups and small and medium-sized enterprises (SMEs): it overcomes a front-loaded investment in IT-related equipment and a life-cycle investment in maintenance, enabling them to use free data and pay for virtual machine capacity as they grow, and rapidly open new applications and markets (Marston, 2011). However, apart from SMEs, larger businesses and governmental agencies require trust as a key factor in the selection process of a platform, as it must align with their operational timeliness, financial stability and security requirements. End users rely on cloud systems to be dependable resources, particularly when these platforms support EO mission-critical applications. They also expect a clear allocation of responsibility and accountability from the providers in the event of issues^100^. Publicly owned platform solutions offer substantial advantages, especially when their governance is designed to offer stability and reliability^101^. CDSE, in fact whose governance is supported by the European Commission and the Copernicus Regulation, fulfils these requirements for a long-term, reliable and openly accessible cloud computing platform^102^. The CDSE contract started in December 2022 initially for six years, with an optional extension to ten years, providing long-term stability to authorities and the user community. The contract is open to future amendments for the purpose of developing new functionality which can be initiated by the user community, the consortium, ESA, or the European Commission.

## Conclusions

By unifying storage, access, and processing under a cloud architecture, CDSE substantially reduces the difficulties associated with downloading, managing, and locally processing EO data. It integrates open standards, APIs, and virtual environments to support the entire EO user community- from beginners to advanced researchers and commercial developers. Through its system’s interoperability such as openEO and standard data access protocols like STAC and OData, CDSE enables automated, scalable and reproducible environmental analysis. The nature of CDSE as an open, publicly owned platform is ensured with its long-term availability and support. Similarly to the way the open availability of Sentinel data has created a revolution in the use of satellite imagery, CDSE can drive the uptake of cloud processing for EO by the masses, empowering new communities that were marginalized by their lack of resources. CDSE is rapidly transforming the world of EO as the first cloud-optimized tool for a global public EO programme.

## Supplementary information

Supplementary material S1

## Data Availability

Datasets composing Copernicus Data Space Ecosystem are available from dataspace.copernicus.eu and various API backends as described in CDSE documentation here: https://documentation.dataspace.copernicus.eu/Data.html.

## Code availability

The code for the openEO API is available on: https://github.com/open-eo. The code for CDSE is available on: https://github.com/eu-cdse. The code for Copernicus Browser (previously Sentinel Hub) is available on: https://github.com/eu-cdse/copernicus-browser.
