# Supplementary material for: Copernicus Data Space Ecosystem establishes public cloud processing for earth observation data
Source: Sci Data. 2026 Feb 26;13:537. doi: 10.1038/s41597-026-06765-8 (PMC13057113; doi:10.1038/s41597-026-06765-8)
Supplement: Supplementary file 1 — Supplementary material S1 [file 41597_2026_6765_MOESM1_ESM.docx]

## Supplementary material S1

Table 1: Data collections available within the CDSE platform

| **Category** | **Mission** | **Purpose** | **Availability** | | | | | |
| --- | --- | --- | --- | --- | --- | --- | --- | --- |
|  |  |  | openEO | Sentinel Hub | STAC | OData | OpenSearch | S3 |
| **Sentinels** |  |  |  |  |  |  |  |  |
|  | Sentinel-1 | C-band SAR imager | X | x | x | x | x | x |
|  | Sentinel-1 Mosaics | S1 temporal composites | x | x | x | x |  | x |
|  | Sentinel-2 | High spatial resolution, 13-band land imager | x | x | x | x |  | x |
|  | Sentinel-2 Mosaics | S2 temporal composites |  | x | x | x |  | x |
|  | Sentinel-3 | Data on ocean and land surface colour, sea and land surface temperature, ocean colour, and sea surface topography | x | x | x | x |  | x |
|  | Sentinel-5P | Atmospheric measurements for air quality, ozone & UV radiation, and climate monitoring & forecasting | x | x | x | x |  | x |
| **Copernicus Services** |  |  |  |  |  |  |  |  |
|  | Copernicus Atmospheric Monitoring Service | Information on atmospheric state and composition |  |  |  |  |  | x |
|  | Copernicus Marine Environment Monitoring Service | Data on physical, sea ice, and biogeochemical state of the marine environment |  |  |  |  |  | x |
|  | Copernicus Land Monitoring Service | Bio-geophysical products on the state and evolution of the land surface on a global scale |  |  |  |  |  | x |
|  | Copernicus Emergency Management Service | Geospatial data and imagery to support decision-making in disaster management |  |  |  |  |  | x |
| **Complementary Data** |  |  |  |  |  |  |  |  |
|  | Copernicus Elevation Models | Earth surface model with vegetation and infrastructure | x | x |  |  | x | x |
|  | Soil Moisture and Ocean Salinity (SMOS) | Passive microwave 2-D interferometric radiometer operating in the L-band |  |  | x | x | x | x |
|  | ENVISAT- Medium Resolution Imaging Spectrometer (MERIS) - decommissioned | Multispectral imager Ocean colour observations, atmospheric- and land-surface-related studies |  |  |  | x | x | x |
|  | Landsat-5 - decommissioned | Multispectral scanning radiometer |  |  | x | x | x | x |
|  | Landsat-7 - decommissioned | Multispectral scanning radiometer |  |  | x | x | x | x |
|  | Landsat-8 | Multispectral scanning radiometer | x |  | x | x | x | x |
|  | Landsat-9 | Multispectral scanning radiometer | x | x | x |  |  |  |
| **Copernicus Contributing Missions (excerpt)** |  |  |  |  |  |  |  |  |
| **Optical sensors** | GeoEye-1 | High spatial resolution, 4-band imager |  |  | x | x |  |  |
|  | GeoSat-1 | Wide-swath, medium spatial resolution multispectral imager |  |  | x | x |  |  |
|  | GeoSat-2 | High spatial resolution, 4-band imager |  |  | x | x |  |  |
|  | Kompsat 3-3A | High spatial resolution, 4-band imager |  |  | x | x |  |  |
|  | PlanetScope | High spatial resolution, 4-band imager |  |  | x | x |  |  |
|  | Pleiades | High spatial resolution, 4-band imager |  |  | x | x |  |  |
|  | Pleiades Neo | Ultra-High spatial resolution, 4 spectral band imager |  |  | x | x |  |  |
|  | Spot 6-7 | High spatial resolution, 4-band imager |  |  | x | x |  |  |
|  | Vision-1 | High spatial resolution, 5-band imager |  |  | x | x |  |  |
|  | WorldView 2-3 | High spatial resolution, 8-band (WV2), 16-band (WV3) imager |  |  | x | x |  |  |
| **Radar sensors** | Cosmo Skymed | Radar altimetry |  |  | x | x |  |  |
|  | Iceye | X-band SAR small satellite constellation |  |  | x | x |  |  |
|  | PAZ | X-band SAR with ultra-high spatial resolution |  |  | x | x |  |  |
|  | Radarsat-2 | Polarimetric imager |  |  | x | x |  |  |
|  | TerraSar-X | X-band SAR |  |  | x | x |  |  |

# **Appendix**

## **Service Quality and level of use**

CDSE is a truly generic platform: at the time of writing, the total registered users are above 500 000 with a gross total of 875 Petabytes (Pb) of downloaded data over the mission’s lifetime. Within the Sentinel missions the most popular satellite is Sentinel-2 with 44 Petabytes of products published on the platform, followed by S1 with 34 Petabytes. S3 and S5P have 6 and 2 Petabytes respectively, within the CDSE platform (<https://dashboard.dataspace.copernicus.eu/>) accessed on 17 November 2025). The most popular data access protocols, are the Process and Catalog APIs, mainly driven by the Copernicus Browser, as the Browser heavily relies on these APIs. Figure 7 depicts a visual overview of the performance statistics and the metrics of user access to the platform.


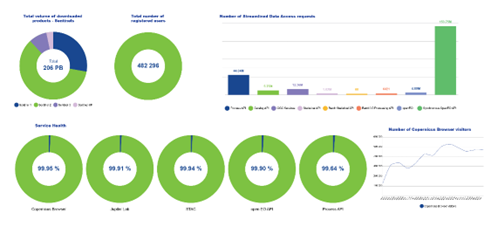


*Figure 7: Overview of CDSE performance and access statistics based on the CDSE dashboard in October 2025*

Since CDSE is empowering public services and industry applications, the reliability of the system is essential. System performance is a result of many different factors in the chain of satellite image acquisition, downlinking, server operation and code maintenance. These factors are monitored and published in the CDSE dashboard. Service availability, measured by PMQ (Potential Maximum Queueing) and PMIA (Potential Maximum Interruption Allowance), averaged 98.7% in 2024 and reached 99.3% in Nov–Dec. Data access endpoints showed 96–99% availability, indicating strong performance.

Since API access is the main driver of CDSE, the number of data access requests is an important metric for its use. In 2025, the typical monthly load is 200-220 million requests, corresponding to 450-475 million processing units. Since one processing unit is about 26 km^2^ of S2 data at full resolution, the total area of data processed by CDSE users through streamlined access every month amounts to about 80 times the area of the Earth’s landmass.

For CDSE as a public infrastructure, usage by non-specialists is just as important as operational data access for downstream solutions. For new or junior users, Copernicus Browser is the main tool for accessing Sentinel satellite imagery. Currently, Copernicus Browser has more than 500 000 monthly visitors.


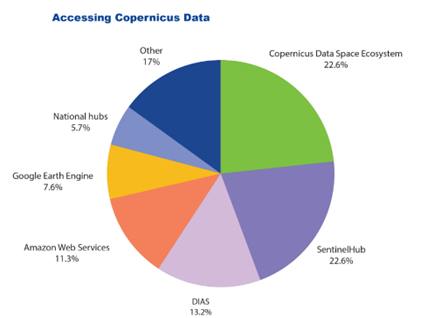


*Figure 8: Frequency of various access options to Sentinel data by European industry companies and government agencies. Based on the EARSC Industry Survey 2024 (European Association of Remote Sensing Companies, 2024)*

In the recent EARSC industry survey (European Association of Remote Sensing Companies, 2024), European companies were asked how they access Copernicus satellite data (Figure 8). 22.6% indicated that CDSE was their main access point, while similarly, 22.6% chose Sentinel Hub. Given that Sentinel Hub is part of the CDSE ecosystem as a service provider, this means that in the 2 years since the platform was launched, it has become the most widespread data access system for Sentinel data in Europe. CDSE recently conducted its second user survey (in April 2025), revealing highly positive feedback: 30% of users reported being “extremely satisfied”, 40% “very satisfied” and 75% indicated that no additional features or services are needed to meet their requirements (Clarijs, 2025), indicating the EO community’s acceptance and tendency to move cloud-based solutions.

Finally, the rollout of CDSE is showing an impact on other commercial and public raster data providers. If users become accustomed to API access as the new paradigm, they will expect to use similar tools to access data from different endpoints. CDSE is creating the critical mass for this with its wide user base. Furthermore, AIRBUS’ Pleiades Neo ultra-high-resolution data available on UP42 is also offered via the API approach. This is expected to enable better data integration and more efficient access for the EO industry in general.
